# Person-Centered Practice in Hospitalized Older Adults with Chronic Illness: Clinical Study Protocol

**DOI:** 10.3390/ijerph191711145

**Published:** 2022-09-05

**Authors:** Diana Alves Vareta, Filipa Ventura, Carlos Família, Célia Oliveira

**Affiliations:** 1 PhD Program, University of Lisbon (UL) and Nursing School of Lisbon (ESEL), 1600-214 Lisboa, Portugal; 2 Egas Moniz Interdisciplinary Research Centre (CiiEM), Egas Moniz Universitary Institute, Quinta da Granja, 2829-511 Monte de Caparica, Portugal; 3 The Health Sciences Research Unit: Nursing (UICISA:E), Nursing School of Coimbra (ESEnfC), 3000-076 Coimbra, Portugal; 4 Laboratory of Molecular Pathology and Forensic Biochemistry, Egas Moniz Universitary Institute, Quinta da Granja, 2829-511 Monte de Caparica, Portugal; 5 Nursing School of Lisbon (ESEL), 1600-096 Lisboa, Portugal

**Keywords:** patient-centered care, aged, chronic disease, inpatients, multimethod studies

## Abstract

The aging trend in the population, the high rate of hospitalization, the affliction by multiple chronic illnesses, and the increased vulnerability of older people when hospitalized undoubtedly require a person-centered approach to healthcare—an approach that values a person’s participation in the healthcare relationship, supports shared decision making and mutual understanding, and respects a person’s values, preferences, and beliefs. However, despite widespread recognition that the adoption of such a clinical practice paradigm is paramount, its implementation and development are still challenging for various health systems and professionals worldwide. The implementation strategy for such a healthcare paradigm must be based on each country’s health system organization and practice contexts, as well as the professionals involved. The present work aims to provide guidelines for the understanding of the state of development of person-centered practice in the daily care of hospitalized older adults with chronic illnesses within the internal medicine department of a secondary hospital in an urban area of Portugal. We focus on the characterization of (i) the perceptions of a multidisciplinary team working at an inpatient hospital department of person-centered practice, (ii) the perceptions of hospitalized older adults with chronic illnesses about person-centered practice, (iii) the work culture of an inpatient hospital department with a high prevalence of older adults with chronic illnesses, (iv) the Person-Centred Practice Framework at the organizational and structural levels of the healthcare system, and (v) the elements that influence the implementation of person-centered practice at the individual, organizational, and structural levels in this specific hospital context. To this end, a mixed-methods analysis with a convergent design was planned to use questionnaire instruments to collect data in parallel and independently from distinct samples of health professionals and older inpatient adults within this department. Furthermore, health policies and strategic plans will be analyzed to identify and evaluate references and guidelines for the practice of person-centered care. Studying the dimensions of clinical practice in this specific healthcare context following the Person-Centred Practice Framework can allow us to understand the extent of its development in terms of prerequisites, care environment, care processes, and the macro-context of the healthcare system. Therefore, it is possible to identify and characterize the dimensions achieved and those that need to be improved and, thus, establish a starting point for the definition of new strategies to advance practice towards person-centeredness and monitor changes in healthcare practice.

## 1. Introduction

Person-centered care is recognized as an approach of great relevance to health systems that supports the restructuring of health policies internationally [1,2,3]. The WHO defines the concept of person-centered care as “an approach to care that consciously adopts individuals’, carers’, families’ and communities’ perspectives as participants and beneficiaries of trusted health systems that respond to their needs and preferences in humane and holistic ways” [3] (p. 5). However, despite widespread recognition of this approach’s importance, several challenges concerning its implementation and development that compromise its adoption into routine healthcare have been reported [4,5,6]. According to the WHO [3], each country should design a strategy for implementing and developing person-centered care according to health system characteristics, care context, and the professionals involved. For this to occur, it is necessary to understand the care practice and the work culture of the healthcare context, as well as to identify the aspects valued by the healthcare team and patients and the potential areas for improvement, always considering the principles of person-centeredness [6,7,8,9].

Person-centered healthcare answers the general population’s needs throughout their life courses. However, elderly people have specificities reinforcing the demand for person-centeredness in care processes [3]. Alongside aging, biological, social, psychological, and cognitive frailties may occur, which trigger functional limitations and difficulties in the ability to respond or adapt [10]. Furthermore, aging is associated with an increased prevalence of chronic illnesses [10]. Older adults with chronic diseases are the population group that uses health services most frequently, seeks hospital care the most, and has recurrent hospitalizations [10,11]. Because of their adaptation challenges, risk of complications, and increased vulnerability, hospitalization might not result in an absolute benefit [10,12].

Considering the presented problem and the importance of contributing to the development of person-centered care in hospital settings, especially for older adults with chronic illnesses, research in this study area is essential. Such works can guide the implementation or the improvement of existing person-centeredness care processes.

The Person-Centred Practice Framework (PCPF) [6] is chosen for theoretical support, as it guides the implementation of person-centered care in practice. The framework integrates the fundamental dimensions of person-centered care to be carried out and covers all levels of care provision, constituting a reference for its implementation and development [6].

### Objectives

The current clinical study protocol is designed to answer the main research question: What is the state of development of person-centered practice in the daily care of hospitalized older adults with chronic illnesses? The state of development refers to the extent to which person-centered practice is found in the context under study, considering that all principles and dimensions presented in the Person-Centred Practice Framework are fundamental for this care practice implementation. To this end, the research protocol is designed explicitly to:

(i) Characterize the perceptions of a multidisciplinary team working at an inpatient hospital department of person-centered practice;
(ii) Characterize the perceptions of hospitalized older adults with chronic illnesses about person-centered practice;
(iii) Characterize the work culture of an inpatient hospital department with a high prevalence of older adults with chronic illnesses;
(iv) Analyze the Person-Centred Practice Framework at the organizational and structural levels of the healthcare system;
(v) Identify the elements that influence the implementation of person-centered practice at the individual, organizational, and structural levels in the hospital context under study.

## 2. Methods

### 2.1. Design

A mixed-methods study with a convergent design will be performed from September 2021 to May 2023. Convergent mixed-methods studies comprise the use of both quantitative and qualitative data from multiple sources regarding research phenomena that may contribute to the comprehensive understanding of a topic [13]. In the established convergent design, quantitative, descriptive, cross-sectional studies (i) and (ii), as well as qualitative descriptive studies (iii) and (iv) will be carried out simultaneously.

Data collection and analysis will take place individually, with the merging of the quantitative and qualitative strings occurring immediately before the statistical analysis of the numerical data and the qualitative analysis of the textual data. Subsequently, the results will be interpreted through a side-by-side comparison using a matrix where coherence and discrepancies between the findings will be identified and analyzed in a concomitant triangulation strategy [13]. The analysis of these findings can allow us to identify elements that influence the implementation of person-centered practice at the individual, organizational, and structural levels in the hospital context under study based on the PCPF (v). Equal weight will be given to the qualitative and quantitative data.

### 2.2. Population and Recruitment

The study will occur at the inpatient internal medicine department of a secondary hospital in an urban area of Portugal. The secondary hospital in Portugal has an area of direct influence for services of up to 500,000 inhabitants, with medical and surgical services in internal medicine, neurology, pediatrics, psychiatry, general surgery, gynecology, orthopedics, anesthesiology, radiology, clinical pathology, immunochemotherapy, and rehabilitation [14]. The internal medicine department of this particular hospital has 55 inpatient beds, and its activity is dedicated to providing care to elderly people with medical conditions, typically multiple chronic diseases. The multidisciplinary team comprises the clinical staff, which entails 4 senior graduate assistants, 12 graduate assistants, 18 assistants, and 20 specific-training interns; the nursing staff, comprised of 50 general care nurses and 3 specialist nurses; and 2 physiotherapists.

Convenience sampling methods will be followed to recruit nurses, physical therapists, and physicians from the healthcare team working at the department, as well as older admitted adults fulfilling the defined inclusion criteria. The healthcare professionals will be the same in the qualitative and quantitative strings since both studies will be developed in the same context, i.e., the internal medicine department of the secondary hospital. The older adults can differ in the qualitative and quantitative studies, given that the number of observations in study (iii) will depend on the saturation of the findings. We anticipate that carrying out study (iii) will require a more extended period and, consequently, cover a different sample of older adults than study (ii).

### 2.3. Inclusion Criteria

Full-time healthcare workers at the inpatient internal medicine department will be recruited for at least six months in order to gather perspectives from the multidisciplinary team on person-centered care practice.

Inpatient older adults over 65 will be eligible to participate in the study of the users’ perspectives if they have a chronic disease diagnosis, are hospitalized at the inpatient internal medicine department, can understand and speak Portuguese, and have no cognitive impairment identified through a 6-Item Cognitive Impairment Test [15].

### 2.4. Exclusion Criteria

Health professionals from other departments or specialties who provide occasional care in the context under study will be excluded from the study.

Elderly people who present clinical instability during data collection, are transferred to other inpatient services, or are discharged in less than 48 h will be excluded.

### 2.5. Sample Size

This study will approach the population of healthcare professionals composing the healthcare team at the inpatient internal medicine department (*n* = 109). Concerning the perspectives of older adults on person-centered practice, study (ii) will have to recruit at least 35 persons to have a minimum of 30 participants, considering a dropout rate of 10–15% [16]. In this case, the variable measured is the person-centered process.

To characterize the workplace culture, the number of observations constituting the sample size will depend on the saturation of the findings [16,17]. Other studies with similar approaches have required between 6 and 20 h of participant observation divided into sets of 15–90 min [18,19].

### 2.6. Data Collection

Data collection will be performed through Person-Centred Practice Inventories that examine how person-centered practice is perceived [20]. The version of the Person-Centred Practice Inventory for health professionals (PCPI-S) [20] will characterize the perceptions of the multidisciplinary team working at the inpatient hospital department on person-centered practice (study (i)). The version for service users (PCPI-SU) [21] will be used to characterize the perceptions of hospitalized older adults with chronic illnesses about person-centered practice (study (ii)). Lastly, the Workplace Cultural Critical Assessment Tool (WCCAT) [17] will allow the characterization of the work culture of an inpatient hospital department with a high prevalence of older adults with chronic illnesses (study (iii)).

All the instruments were translated into Portuguese and culturally adapted. The preliminary psychometric evaluations of the PCPI-S and PCPI-SU showed that these translated versions reflected the original measurement structure, with good fit indices. Nonetheless, the instruments will be applied to the subjects in this study when scientific reports of the translated versions are only under peer review.

In addition to the PCPI-S and PCPI-SU, socio-demographic data and the professional characterizations of the healthcare team will be performed. The PCPI-S will be delivered to the healthcare professionals through Google Forms^®^ (Google Corp. 2018, Dublin, Ireland) using an institutional email to ensure the anonymity of the information obtained. The PCPI-S is a self-reported instrument composed of 59 items on a 5-point Likert-type response scale, with higher values indicating higher agreement [20]. The PCPI-S measures three dimensions derived from the PCPF (i.e., prerequisites, care environment, and care processes) and comprises 17 constructs. These are professional competency, developed interpersonal skills, self-knowledge, clarity of beliefs and values, commitment to job, appropriate skill mix, shared decision-making systems, effective staff relationships, power sharing, physical environment, supportive organizational systems, potentials for innovation and risk taking, working with patients’ beliefs and values, sharing decision making, engaging authentically, being sympathetically present, and providing holistic care [6]. The instrument has been reported to be valid and reliable in other studies [22,23,24].

The PCPI-SU will be administered to older adults by the study’s principal researcher, anticipating challenges in interpreting or filling out the inventory. The PCPI-SU is a 5-point Likert scale with 20 items that assesses the levels of agreement of patients with statements about the care process dimensions described in the PCPF and comprises 5 constructs. These are working with patients’ beliefs and values, sharing decision making, engaging authentically, being sympathetically present, and providing holistic care. The instrument has been tested for face validity and, subsequently, translated, as well as tested into Danish and German, but these studies are not yet published [21].

Participant observation will be guided by the WCCAT, which systematically obtains data from observing the interactions between participants and the context [17]. The WCCAT is an instrument to support the observation of practice that integrates the main constructs of prerequisites, care environment, and care processes [25]. The International Community of Practice members reviewed it to ensure its alignment with the current PCPF and further tested it for face validity [17].

Finally, a documentary analysis of Portuguese health policies and strategic plans (primary sources) will be carried out to identify references and guidelines for the practice of person-centered care. Documents relating to the study’s hospital and the department will also be included, focusing on organizational structure, mission and objectives, principles of good governance, and leadership strategy. Documents published from 2012 to 2022 will be considered. The year 2012 was defined as the research baseline, as the 2012–2016 National Health Plan contemplated for the first time that the provision of person-centered healthcare was a standard to be followed in the provision of healthcare in Portugal [26]. A data collection tool will be developed within the research team and validated by experts in person-centered care. The instrument categories will be defined based on the components of the PCPF [6].

### 2.7. Statistical Data Analysis

Quantitative data will be treated using a statistical package for social sciences software (IBM SPSS Statistics^®^ for windows, v. 27.0. IBM Corp. Released 2020, Armonk, NY, USA). A descriptive analysis (i.e., mean, standard deviation, minimum, and maximum) of the constructs that compose the PCPI versions will be performed.

Subsequently, an analysis of variance (ANOVA) will be performed to determine the effect of different demographic and professional characterization variables on the questionnaire constructs described in the previous section. Demographic and professional characterization variables with more than two levels that are shown to influence the construct under analysis significantly will be further evaluated using Tukey’s post hoc test for multiple comparisons. The model assumptions will be assessed by analyzing a plot of residuals versus predicted values, a Q–Q plot of residuals, and a residual histogram. Whenever a violation of an ANOVA assumptions occurs, the dependent variable or construct will be transformed using a Box–Cox transformation. After this, a new ANOVA will be conducted, and the assumption will be reverified as described above.

The care process concordance levels between the two groups (patients and health professionals) will be compared using Student’s *t*-test or alternative nonparametric tests. The test choice will depend on whether the data is normally distributed (Shapiro–Wilk test) and if there is a homogeneity of variances between groups (Levene test).

### 2.8. Thematic Content Analysis

Qualitative data analysis will be carried out through a thematic content analysis in an interpretive and iterative process of synthesizing the relevant data to answer the research question, identifying patterns of meaning and classifying thematic units as the data analysis takes place [27,28]. The use of MAXQDA^®^ 2022, (VERBI Software, 2021; Berlin, German) software in data management is foreseen.

### 2.9. Ethical Considerations

The study received ethical approval from the study’s Hospital Ethics Committee (ref. nr. 36/2021). All procedures will be carried out according to the Declaration of Helsinki [29] and will comply with the General Data Protection Regulation [30].

Authorization was requested to use the data collection instruments from the respective authors.

Information about the studies will be provided verbally and in written format to prospective participants prior to data collection, aiming to gather their written informed consents. The information provided will focus on the objective, relevance, data collection methods, expected participation, and dissemination of the research product.

All data collected from the participants will be strictly anonymous and confidential. Only the researchers will have access to all the data. Essential documents will be archived in password-protected files.

Considering that the participants over 65 years old have increased vulnerability [10,12] and are ill and hospitalized, protective measures will be taken. Specifically, a reflection period on participation in the studies of at least one day will be available, and the PCPI-SU will then be administered by the principal researcher, anticipating subjects’ challenges in interpreting or filling out the questionnaire. Participants who receive care will be assured that participation in studies (ii) and (iii) will not influence the care provided, emphasizing that there is no risk of personal consequences arising from the decision to participate.

Health professionals will be guaranteed anonymity and will not be subject to evaluation or penalty given the answers provided in study (i) or behaviors demonstrated in study (iii).

## 3. Discussion

A search on ClinicalTrials.gov, the Center for Open Science, and Google Scholar^®^ was carried out to identify clinical trial protocols to study and characterize the implementation of person-centered practices according to the PCPF model, including the perceptions of those involved in the therapeutic relationship, healthcare professionals, and patients. The fact that no results were obtained reinforced this investigation’s innovative and relevant character. Furthermore, no information was found on developing person-centered care in the daily care practice of Portuguese healthcare departments. Therefore, this study represents a starting point for the characterization and evaluation of the implementation of person-centered practice in Portuguese healthcare.

The relevance of this study is transversal to several disciplines related to healthcare, as person-centered practice results from multidisciplinary interaction [6]. To the best of our knowledge, there are still no published studies on the PCPF from an interdisciplinary perspective. Studying person-centeredness in a healthcare context based on the PCPF can allow us to improve our understanding of the extent of the development of care practice, as it can enable the identification of which components of the model are present at the individual, organizational, and system levels [6,31]. Such a study can allow us to identify and characterize the person-centered practice dimensions achieved and those that need further improvement toward enhancing care quality, patient safety, and satisfaction.

The decision to use this framework was based on the premise that it promotes understanding of the concept through the definition of critical elements and guides its operationalization in practice [6]. The data collection instruments used in this study were derived from the PCPF and have been empirically validated [17,20,21].

The results of this clinical study are expected to impact how healthcare professionals and managers consider person-centered practice in the practice context. Furthermore, applying this research methodology to other healthcare contexts can allow a broad, comprehensive view of person-centered practice in Portugal from a multidisciplinary perspective.

The methodological strategy can also be applied internationally across settings and clinical areas, as it allows for the generation of evidence from comparative studies. Further investigation across settings and larger multiprofessional and patient populations is required to test the effectiveness and adaptability of the strategy design.

Continuity can be given to this study with a research project that explores and monitors person-centered practice according to the PCPF model, including individual, organizational, and structural levels of practice and the perceptions of those involved in the therapeutic relationship.

## 4. Conclusions

In this manuscript, we presented a study protocol, as well as its aims, methodological approaches, and plans, that allowed us to operationalize this research. The findings are expected to directly relate to several healthcare and medical setting policies across Portugal and provide new information that stimulates person-centered care education for healthcare professionals and inpatients.

This study has the potential to contribute to the characterization of the dimensions of person-centered care as they are seen in the current inpatient context to identify which factors need to be improved, therefore establishing a starting point for defining strategies to advance practice toward person-centeredness and monitor changes in healthcare practice.

Nevertheless, we emphasize that the study can have limitations. One possibility is that healthcare participants may favor their perceptions of person-centered care in their work context if they recognize it as socially desirable. Alternatively, if inpatient participants perceive it to be socially desirable, they might overstate their experiences of person-centered care.

## Data Availability

Not applicable.
